# Longitudinal Trends of the Annual Exposure to PM_2.5_ Particles in European Countries

**DOI:** 10.1155/2021/8922798

**Published:** 2021-12-10

**Authors:** Mahdiyeh Alikhani Faradonbeh, Gashtasb Mardani, Hadi Raeisi Shahraki

**Affiliations:** ^1^Student Research Committee, Shahrekord University of Medical Sciences, Shahrekord, Iran; ^2^Department of Environmental Health Engineering, Faculty of Health, Shahrekord University of Medical Sciences, Shahrekord, Iran; ^3^Department of Epidemiology and Biostatistics, Faculty of Health, Shahrekord University of Medical Sciences, Shahrekord, Iran

## Abstract

**Background:**

PM_2.5_ emission is known as a major challenge to environmental health and is the cause of approximately 7 million deaths annually. This study aimed at investigating the main patterns of PM_2.5_ trend changes among European countries.

**Methods:**

The annual exposure to PM_2.5_ pollutants was retrieved from the World Bank for 41 countries during 2010 to 2017, and a latent growth model was applied to identify the main patterns using Mplus 7.4 software.

**Results:**

Monitoring the overall mean annual exposure to PM_2.5_ in the Europe showed a downward pattern with an annual decrease of 2.48% during the study period. Turkey had the highest PM_2.5_ exposure with 43.82 *μ*g/m^3^ in 2010, reaching 44.31 *μ*g/m^3^ in 2017. Likewise, with 7.19 *μ*g/m^3^ in 2010, Finland had the lowest exposure level which decreased to 5.86 *μ*g/m^3^ in 2017. Two main patterns for the mean annual PM_2.5_ exposure were identified via the latent growth model. Countries in the first pattern, including Turkey and Ukraine, had experienced a slow annual increase in the mean exposure of PM_2.5_ pollutant. Likewise, the other 39 countries belonged to the second pattern with a moderate falling trend in the mean exposure to PM_2.5_.

**Conclusion:**

Although the trend changes of mean annual exposure to PM_2.5_ in Europe were falling, Turkey and Ukraine had experienced a slow annual increase. It is advisable to take appropriate measures to curb the current raising exposure to PM_2.5_ in Turkey and Ukraine.

## 1. Introduction

Air pollution causes exposure to toxic substances in the atmosphere which has detrimental effects on human health. Compounds of particulate matters (PMs) vary by time and place and may include metals, ions, organic compounds, quinoid stable radicals, carbon, minerals, reactive gases, and materials of biological origin [1]. The association between exposure to PM_2.5_ and death rate among people aged 45 and over was confirmed by previous studies [2]. The latest reports from the European Environment Agency (EEA) shows that just in 2015, more than 500,000 people died due to air pollution and about 83% of the aforementioned mortalities was due to particulate matter [3].

Monitoring the trend of PM_2.5_ particles emission in Europe indicates that it has been increasing since 1950, peaking from 1980 to 1990 and decreasing from 1990 to 2015 [4]. The results of a study on the concentration of PM_10_ and PM_2.5_ particles in 2012 in 25 European countries show that the average annual PM_10_ in 77% of stations and in all countries (except Estonia and Ireland) exceeded WHO guidelines. Moreover, the annual concentration of PM_2.5_ particles in 89% of control stations was higher than the WHO guidelines [5].

In the period 2013–2017, the concentration of PM2.5 in 12 European countries has changed significantly on various European sites, and the average PM_2.5_ varies from 3.5 *μ*g/m^3^ in Stockholm to 21 *μ*g/m^3^ in Paris. Northern European cities showed lower levels of PM_2.5_ than cities in the southern and central regions. Significant levels of concentration were observed in places in the Mediterranean region, especially in Athens and Istanbul (Southeast Europe) [6].

Despite the importance of the subject matter, most studies have so far been limited in time or geography, and no comprehensive comparison has been made on the trend of particulate matter change in the whole of Europe. Therefore, this study aimed at investigating the main patterns of PM_2.5_ trend changes among European countries in the period 2010–2017.

## 2. Materials and Methods

The annual exposure to particulate matter with an aerodynamic diameter of less than 2.5 *μ*m (PM_2.5_ particles) information based on micrograms per cubic meter was provided from the World Bank website produced for the Global Burden of Disease study, provided by the Institute for Health Metrics and Evaluation at the University of Washington. Ambient PM2.5 was evaluated by annual average PM2.5 concentration in the air, calculated by satellite data, chemical transport models, and ground-level measurements at a spatial resolution of a 0.1 × 0.1 grid [7, 8]. Exposure is calculated by weighting mean annual concentrations of PM2.5 by the population in both urban [9].

Related information for 41 European countries (all the available European countries) was extracted as an Excel file from 2010 to 2017.

The latent growth model was applied to identify the main patterns of PM_2.5_ trend changes in Europe. To estimate each of the *k* latent patterns, the following equations were utilized:

(1) $$\begin{array}{c} y_{it}^{k}=\alpha_{i_{0}}^{k}+\alpha_{i_{1}}^{k}\lambda_{t}^{k}+\varepsilon_{it}^{k},\\ \alpha_{i_{0}}^{k}=\alpha_{00}^{k}+\sum_{j}\beta_{01j}^{k}x_{j}+\varepsilon_{i_{0}}^{k},\\ \alpha_{i_{1}}^{k}=\alpha_{10}^{k}+\sum \beta_{11j}^{k}x_{j}+\varepsilon_{i_{1}}^{k}, \end{array}$$

where the overall mean of PM_2.5_ at 2010 in the *k*th pattern is denoted by *α*_00_^*k*^ and *α*_10_^*k*^ is the mean rate of trend changes in PM_2.5_ for the *k*th pattern. *P* < 0.05 was set as statistically significant in Mplus 7.4 software. To identify the main patterns of PM_2.5_ trend changes, the latent growth models with different number of patterns was fitted. Moreover, the likelihood ratio test (LRT) was taken into consideration to estimate the number of latent patterns.

## 3. Results

**Figure 1:**
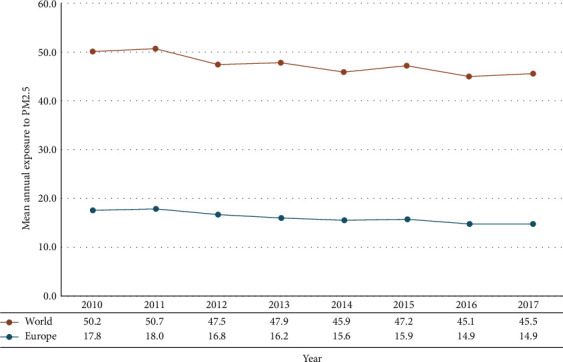
The trend changes of mean annual exposure to PM_2.5_ in the world and Europe.

Monitoring the overall mean annual exposure to PM2.5 in Europe showed a falling pattern with an annual decrease of 2.48% during the study period (Figure 1). Turkey had the highest level of exposure to PM_2.5_, with 43.82 *μ*g/m^3^ in 2010 which reached 44.31 *μ*g/m^3^ in 2017. On the other hand, Finland had the lowest exposure with 7.19 *μ*g/m^3^ in 2010 which decreased to 5.86 *μ*g/m^3^ in 2017.

Goodness of fit indices are summarized in Table 1. Based on LRT, we proposed a model with two patterns. More information about the number of countries, mean exposure at 2010, and mean annual change of each pattern is reported in Table 2.

**Figure 2:**
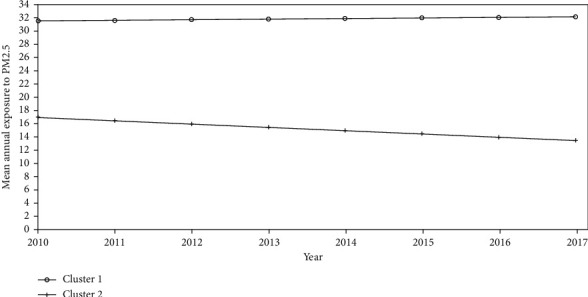
The linear trend of mean annual exposure to PM2.5 in the two identified patterns.

Countries in the first pattern, including Turkey and Ukraine, had experienced a slow annual increase (+0.09) in the mean exposure to the PM_2.5_ pollutant. The other 39 countries belong to the second pattern with a mean exposure at 2010 of 16.9 *μ*g/m^3^ and a moderate falling trend with an annual decrease of −0.50 in the mean exposure to PM_2.5_ (Figure 2).

## 4. Discussion

Modeling the PM_2.5_ exposure trend from 2010 to 2017 in Europe showed that Turkey and Ukraine had a very slow rising trend and the other 39 countries had experienced a moderate descending trend. Although the falling trends of PM_2.5_ and PM_10_ pollutants were confirmed in the NE region in Spain, the observed trend was not constant during 2004 to 2014. The declining share of PM particles, such as street and traffic, as well as the impact of air pollution control measures were the main reasons of decreasing PM_2.5_ and PM_10_ exposure [10]. Another study in the Castellon region of Spain approved the falling trend of PM_10_ particles concentration in urban and industrial areas due to the economic crisis, which led to a decline in industrial production [11]. Investigation of temporal changes in the annual concentration of PM_2.5_ in Serbia showed a falling trend from 2001 to 2016 [12]. In Georgia, a 33% decrease in the total PM_2.5_ mass was reported during the period of 2002 to 2013 due to the implementation of pollution control policies such as reducing fuel coal combustion [13].

Examining the long fashion trend of PM_2.5_ particles in Augsburg (Germany), Brisbane (Australia), London (UK), Rochester (USA), and Helsinki (Finland) confirmed a uniform trend with a negative slope in all the cities from 2001 to 2017. Despite population and economic growth, the decline in PM concentration was the result of controlling pollution control measures [14]. Moreover, a comprehensive assessment of air pollution in the UK showed a steady decline in PM_2.5_ particle concentration from 1970 to 2010 [15]. Regular updates in the fleet (introduction of vehicles with more efficient engines) as well as the economic crisis that began in 2008 and led to the shutdown of many industries producing PM_2.5_ were identified as the main reasons for the observed falling trends of PM_2.5_ and the annual concentration of PM_10_ particles during the period 2002–2015 in Italy [16]. In Krakow (Poland), despite the doubling of the fleet size, PM_10_ and PM_2.5_ particles decreased by 39% and 35%, respectively, from 2010 to 2015. This was due to the replacement of Euro VI engines for Euro IV engines which reduced the emission of these particles [17]. In Greece, declining trends of PM_2.5_ and PM_10_ were reported as 58% and 52%, respectively, from May 2008 to April 2013. This is mainly due to the decrease in urban emissions, antipollution measures along with the reduction of industrial activities, and transportation during the financial crisis [18]. An increase of about 27% in the share of Euro engines in vehicles and, on the other hand, the prevalence of initiatives to use the combination of LPG/gasoline, hybrid, and electric vehicles contributed greatly to the reduction of PM_2.5_ emission in the Malta Islands [19].

Investigation of the pollutants' concentration in Turkey showed no significant difference in the concentration of PM_10_ and PM_2.5_ particles between 2016 and 2017. However, their values were higher than the WHO standard, which is in line with the results of the current study. Since the correlation between PM and temperature particles for suspended particles in hot seasons causes a positive trend and a negative trend in cold seasons, PM particles' concentration is constant in this region. The reason is the uniformity of wind speed and direction in that area and the balance in climatic conditions. Besides, the strong correlation between PM_2.5_ and CO indicates the higher contribution of vehicle sources in pollutant concentrations [20]. In line with our findings, investigation of the PM_10_ concentration in Ankara showed a moderate increase from 36 *μ*g/m^3^ in 2007 to 77 *μ*g/m^3^ in 2013 [21]. The raising trends of PM_10_ and PM_2.5_ pollutants in Turkey was also confirmed by Çapraz et al. who found that the increase in the number of respiratory patients in the period of 2013–2015 was associated with an increase in concentrations of PM_10_ and PM_2.5_ pollutants [22]. The increasing longitudinal trends of PM_2.5-10_ and PM_2.5_ concentrations in Chernivtsi (Ukraine) from 2013 to 2014 were approved in the study of Lanzinger et al. Their study showed that there was a positive association between the concentration of PM particles and cardiovascular disease mortality [23]. The increasing trend of PM_2.5_ in Ukraine also was reported in a study by Nekos et al. during the period of 2014 to 2016 [24].

## 5. Conclusions

There is no doubt that particulate matters less than 2.5 microns are one of the most important causes of death. Although the trend changes of mean annual exposure to PM_2.5_ in Europe was falling, Turkey and Ukraine had experienced a slow annual increase. It is advisable to perform urgent action to control the current rising trend of exposure to PM_2.5_ in Turkey and Ukraine.

## Figures and Tables

**Table 1:** The summary of goodness of fit indices for different number of patterns.

Fit indices	Number of patterns					
	1	2	3	4	5	6
AIC	818	751	750	743	741	743
BIC	841	782	790	791	797	808
LRT *P* value	—	0.001	0.60	0.17	0.18	0.16

AIC: Akaike information criterion; BIC: Bayesian information criterion.

**Table 2:** The number of countries and mean annual change for each pattern.

Pattern	Number of countries	Mean exposure at 2010		Mean annual change	
		Estimate	SE	Estimate	SE
First	2	31.47	6.52	0.09	0.43
Second	39	16.95	1.02	−0.50	0.04

## Data Availability

The data are freely available at the Gapminder website (https://www.gapminder.org/data/).
